# A finite element study for tibial fractures: analyze the biomechanical condition of the tibial fracture area to provide guidance for subsequent treatment

**DOI:** 10.3389/fbioe.2025.1532207

**Published:** 2025-06-20

**Authors:** Ke-Rui Zhang, Bin Luo, Ju Tu, Ya-Qin Li, Jun Wen, Chang-Yong Shen, Xue-Hai Jia, Yi Deng, Li-Tai Ma, Yi Yang

**Affiliations:** ^1^ Department of Orthopedics, Orthopedic Research Institute, West China Hospital, Sichuan University, Chengdu, China; ^2^ Department of Orthopedics, Ya’an People’s Hospital, Ya’an, China; ^3^ School of Nursing, the Hongkong Polytechnic University, Hongkong, China; ^4^ Department of Computer Science and Technology, Southwest University of Science and Technology, Mianyang, China

**Keywords:** intramedullary nails, STRESS value, deformation value, displacement value, sensor, finite element analysis

## Abstract

**Introduction:**

Distal tibial fractures are common fracture sites and usually require surgical treatment to achieve anatomical reduction. Intramedullary nails (IMN) are widely used in orthopedics for stabilizing fractured bones and treating limb deformities. The process of postoperative bone healing is of great significance for patient rehabilitation and can guide subsequent treatment methods. However, the current radiographic techniques used to determine the degree of fusion, such as X-ray, need to be improved in accuracy and have some radiation effects. Several studies suggested that the mechanical load on the fracture area could reflect the bone healing process and evaluated the stability of fracture area. The aim of this study is to investigate the biomechanical changes in the fracture area during bone healing and IMN, and to prepare for the subsequent placement of intelligent stress and displacement sensors based on the changes in stress and displacement, in order to provide guidance for the treatment and rehabilitation of postoperative fractures.

**Methods:**

Finite element (FE) models representing different healing stages of tibial fractures were developed. All conditions were applied to simulate the stress and strain of the IMN fixation system under normal tibial stress.

**Results:**

The stress at the fracture area on the IMN gradually decreases, while the stress on the callus gradually increases until reaching a stable state at the 12th week after surgery. And the deformation value and the displacement value of the callus decrease and stabilize over time. Based on the changes in stress at the fracture area of the IMN and the displacement value of the callus, we can place a stress sensor at the fracture area of the IMN and a displacement sensor at the callus area.

**Conclusion:**

This study utilized FE analysis to evaluate stress, deformation and displacement between the IMN and bone during the healing process of tibial fractures in four stages. By combining these aspects, the degree of bone healing can be assessed. This research enables orthopedic doctors to monitor the progression of fracture healing without relying solely on imaging examinations. Furthermore, it aids in guiding patients to undergo appropriate rehabilitation training for better recovery.

## Introduction

The tibia is a crucial load-bearing bone in the human body, located on the inner side of the calf and is more prone to fractures (Wildemann et al., 2021). Especially the distal tibial fractures are common fracture sites and usually require surgical treatment to achieve anatomical reduction. Intramedullary nails (IMN) osteosynthesis is a widely used surgical method for treating extra-articular tibia fractures (Chen et al., 2018), because of its lower rates of infection and less soft tissue dissection (Meena et al., 2015).

Postoperative monitoring of bone healing is crucial as it signifies the patient’s functional recovery. Currently, the standard methods for surgeons to examine the degree of bone healing relies heavily on traditional imaging examination methods, such as X-rays (Lack et al., 2014). However, radiographic evidence is an indirect method for fusion assessment and may not adequately reflect the extent of bone healing. The reason is that the X-rays provide intermittent measurements and cannot reflect real-time healing status. Frequent X-rays can also pose radiation risks and economic burdens for patients. Additionally, X-rays have low spatial resolution and may not clearly identify small lesions or thick areas, especially during the early stages of healing when cartilage formation is critical for bone healing assessment. Unfortunately, X-ray does not clearly recognize the early formation of cartilage.

This study aims to address these issues by monitoring indicators like stress value, deformation value, and displacement value of the fracture area. By monitoring changes in stress values of the fracture area, we can establish a relationship between bone healing stage and stress values. And stress sensors are installed at locations on the fixation system, based on changes in stress values. By monitoring deformation and displacement values, a relationship between the two and the process of bone healing can also be established. At the same time, the position of the displacement sensor can also be determined. The numerical changes of the stress and displacement sensors mentioned above can be used to determine the process of bone healing, and provide an advanced and convenient method for clinical evaluation of bone healing. This information also helps to provide patients with appropriate postoperative rehabilitation training.

FE analysis is a vital method for evaluating stress in different parts of the human skeleton. By adjusting FE settings, such as material properties and boundary conditions, this method provides biomechanical evaluation and prognosis for various diseases, injury types, implant fixation, and surgical techniques (Abdul Wahab et al., 2020). In this study, a FE model was established based on the tibia of a healthy adult using IMN fixation systems at 1 week, 4 weeks, 12 weeks, and 90 weeks after tibial fracture. The stress states of the tibia and IMN during different bone healing stages under physiological conditions were simulated. The study explored stress value, deformation value, and displacement value of the fracture area, providing a arithmetic basis for subsequent sensor placement.

## Materials and methods

The tibia in the three-dimensional FE model was derived from the CT images of a healthy male individual. CT scans with a thickness of 0.75 mm and an interval of 0.69 mm were acquired using a CT scanner (SOMATOM Definition AS+, Siemens, Germany). The male participant had a height of 173 cm, a weight of 65 kg, and did not have any relevant conditions such as osteoporosis, osteoarthritis, or fractures. The IMN fixation used in this study was sourced from America (Johnson & Johnson), United States, which is composed of nails and its attachments.

### Experimental model

**FIGURE 1:**
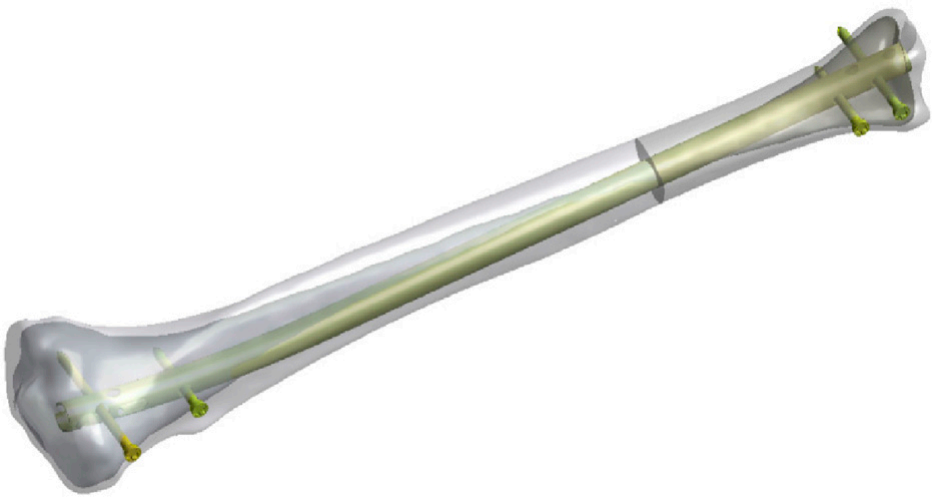
The creation of fracture model and fixation implements used in this study.

Model Creation: Firstly, the CT data of the healthy volunteer’s tibia were saved in Dicom format and imported into Mimics 19.0 (Materialize Inc., Leuven, Belgium). Separate STL models of the tibial cortical bone and trabecular bone were created to accurately represent the internal and external structure of the bone. Subsequently, these models were imported into Geomagic Studio 14.0 software (3D Systems Inc., Rock Hill, SC, United States) for model smoothing and solidification processing to reduce model complexity while ensuring completeness and accuracy. At this stage, a three-dimensional model of the external fixation apparatus was created based on the actual sample. The models processed as described above were exported in STP format and further imported into Solidworks 2021 software (Dassault Systemes S.A, United States). A 3 mm transverse defect was created 13 cm from the distal end of the tibia to simulate a transverse fracture in the middle and lower 1/3 of the tibia (AO type 42A3). Then, the IMN system was assembled with the tibial model, with four screws symmetrically distributed on both sides of the fracture end. All screws made contact with the opposite cortex but did not penetrate it, as shown in Figure 1. After completing the model assembly, an interference check was performed to ensure there were no overlapping areas, thus avoiding computational errors in subsequent calculations.

### FE model

The complete model of tibial fracture fixation surgery was imported into Ansys Workbench 2022 R1 (ANSYS WORKBENCH, ANSYS. Software Corporation, Canonsburg, United States) for FE meshing and analysis. All models were set as uniform and linearly isotropic material properties and meshed using first-order tetrahedral elements. An iterative solver type was selected for the setup. During the configuration, the overall mesh density was set to 3mm, with a 1 mm mesh refinement applied to the fixation devices, contact areas, and callus areas to enhance analysis precision. In actual surgery, screws are closely fixed to the bone, and the internal components of the external fixation device provide strong stability. Therefore, bonding contact was defined between them. Following previous research (Di Puccio et al., 2021) (Young’s moduli were set to 0.007 GPa, 0.13 GPa, 10.02 GPa, and 16.7 GPa for postoperative weeks 1, 4, 12, and 90, respectively, with a Poisson’s ratio of 0.26 for all stages), this study defined all materials, including bone and the external fixation device, as homogeneous and linear isotropic, Young’s moduli were set to 0.007 GPa, 0.13 GPa, 10.02 GPa, and 16.7 GPa for postoperative weeks 1, 4, 12, and 90, respectively, with a Poisson’s ratio of 0.26 for all stages. The material properties and mesh types used in this study are presented in Table 1, while the node and element counts for the tibia, callus, and fixation device are presented in Table 2.

**TABLE 1:** Material properties and mesh types.

	Modulus of elasticity (GPa)	Poisson’s ratio	References source	Element type
Cortical bone	16	0.3	Abdul Wahab et al. (2020)	Shell elements
Cancellous bone	1.1	0.26	Abdul Wahab et al. (2020)	3-D solid elements (4 node)
Screw	110	0.3	Chen et al. (2018)	3-D solid elements (4 node)
Intramedullary nailing	110	0.3	Chen et al. (2018)	3-D solid elements (4 node)
Fracture end tissues (Week 1)	0.007	0.26	Di Puccio et al. (2021)	3-D solid elements (4 node)
Fracture end tissues (Week 4)	0.13	0.26	Di Puccio et al. (2021)	3-D solid elements (4 node)
Fracture end tissues (week 12)	10.02	0.26	Di Puccio et al. (2021)	3-D solid elements (4 node)
Fracture end tissues (week 90)	16.7	0.3	Di Puccio et al. (2021)	3-D solid elements (4 node)

**TABLE 2:** The number of nodes and elements in each part.

	Number of nodes	Number of elements
Tibia	145,700	88,605
Fracture end tissues	12,452	7,729
Intramedullary nail	176,243	89,103

### Loading and boundary conditions

**FIGURE 2:**
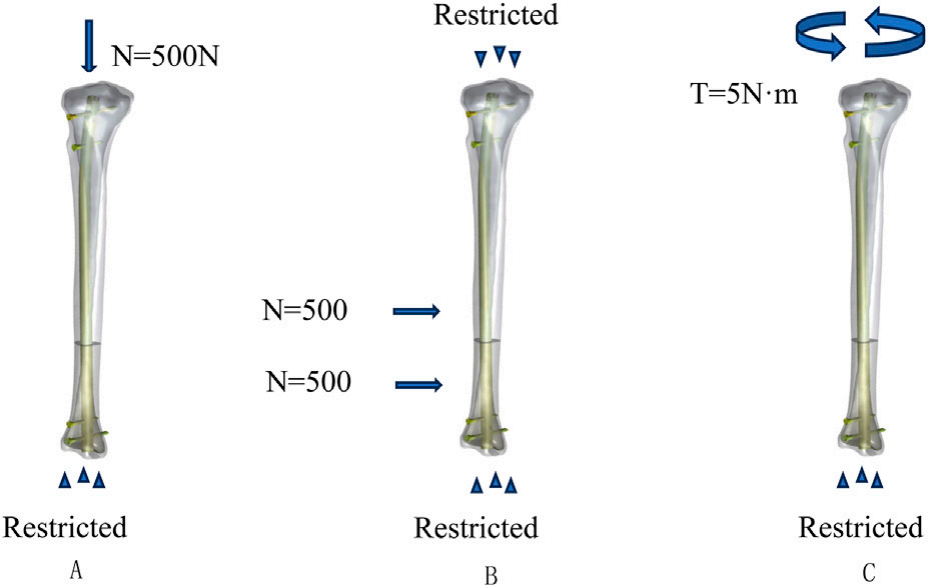
Loading and boundary condition: **(A)** loading and boundary conditions for compression, **(B)** loading and boundary conditions for four-point bending, and **(C)** loading and boundary conditions for torsion.

In this study, we designed three different loading conditions: axial, torsional, and four-point bending. Previous research has indicated that the tibial plateau experiences a force equivalent to 70% of body weight when a person is standing and doubles when in a walking state compared to standing (Shark, 2010). Therefore, we defined the axial vertical load as 500N with the tibia’s distal end fixed. Under torsional loading conditions, the tibia’s distal end was fixed, and a counterclockwise torque of 5Nm was applied at the proximal end of the tibia. With both the proximal and distal ends of the tibia fixed, 500N loads were applied at a distance of 36 mm from the fracture line to simulate four-point bending loads (Li et al., 2018), as shown in Figure 2.

## Results

### Stress value and its variation at fracture area

**FIGURE 3:**
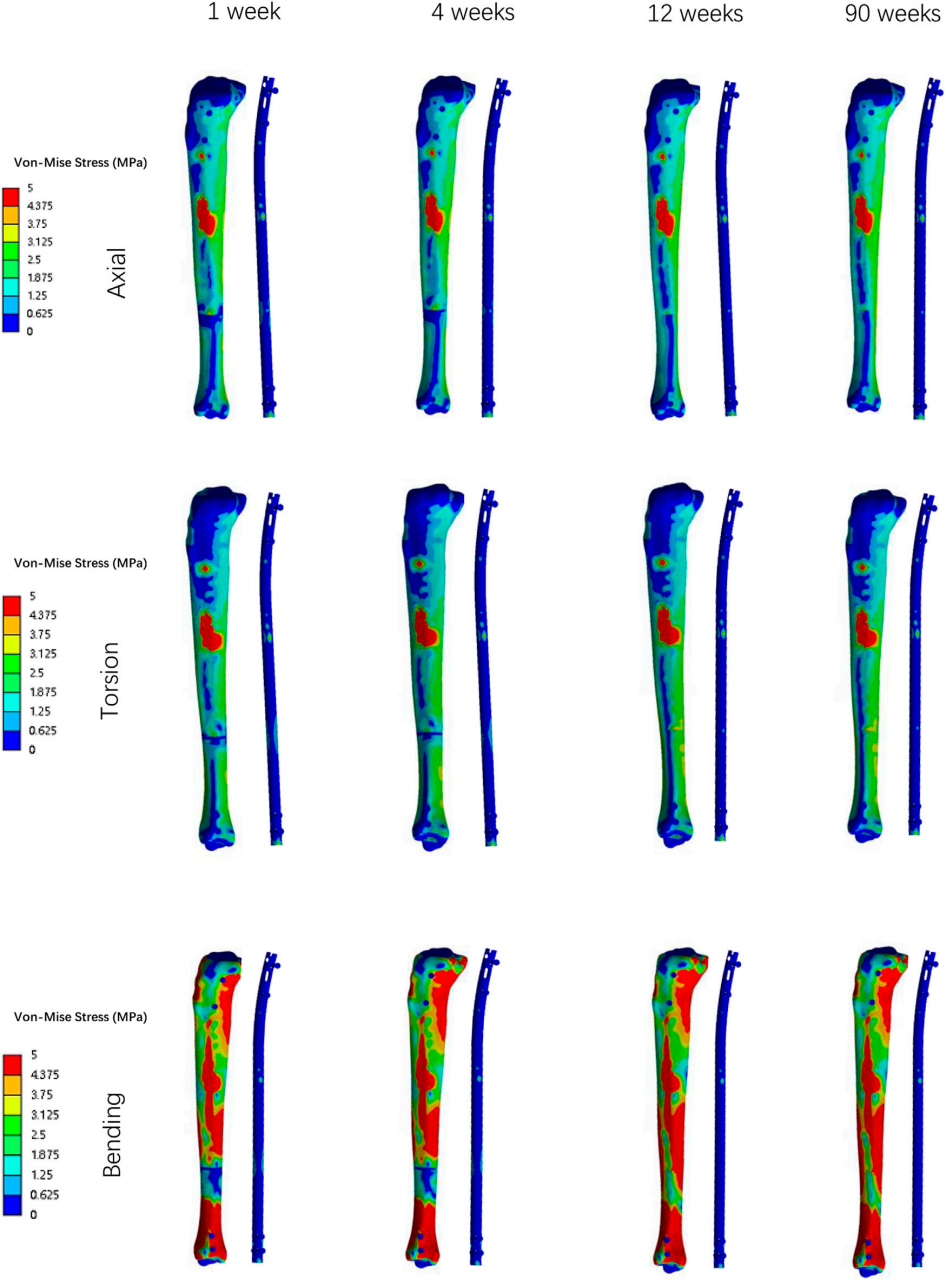
The Von Mises stress distribution on the surfaces of tibia and IMN system in all loading conditions in different stages of bone healing.

**FIGURE 4:**
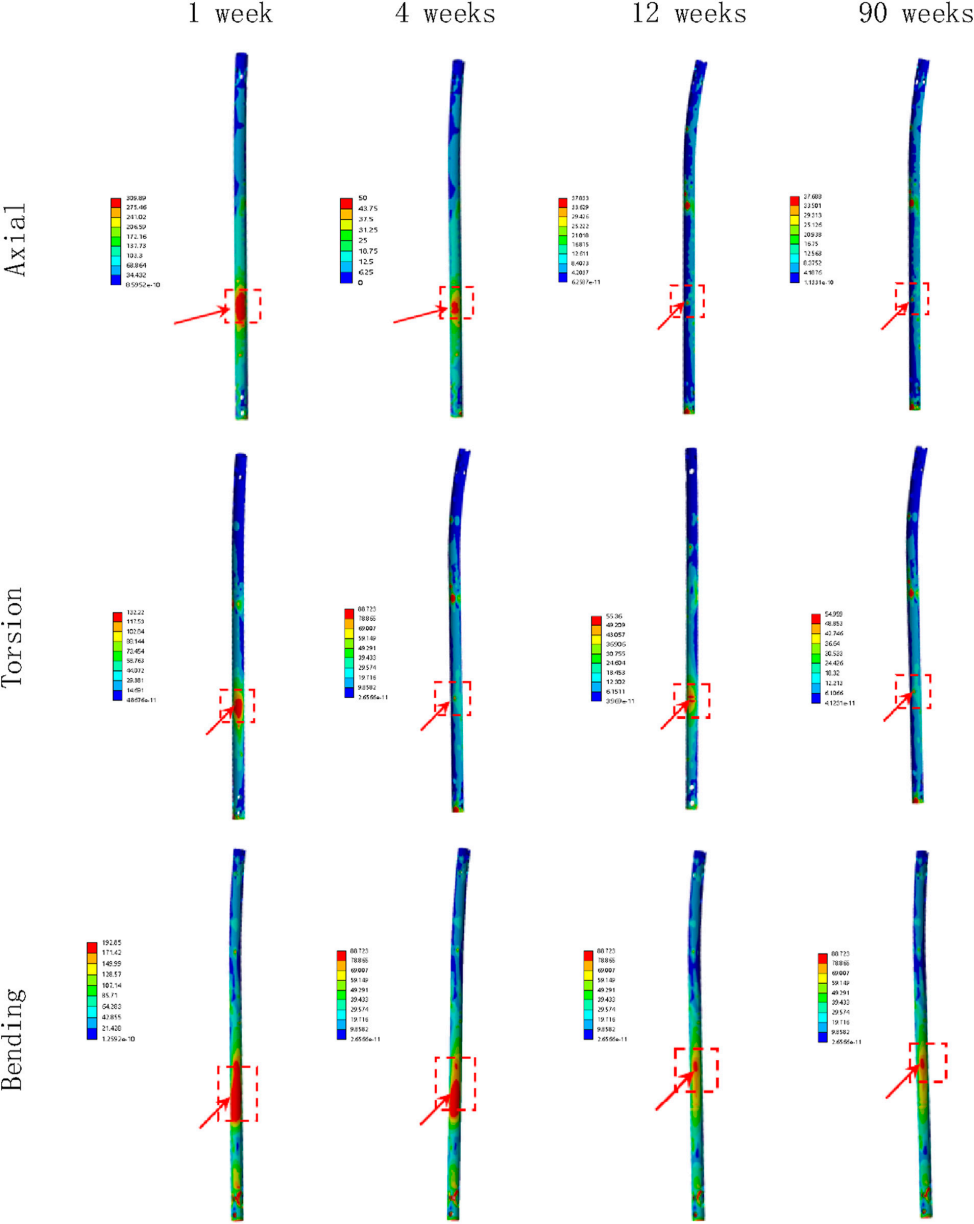
The stress cloud map of the IMN at the fracture area in all loading conditions in different stages of bone healing.

**FIGURE 5:**
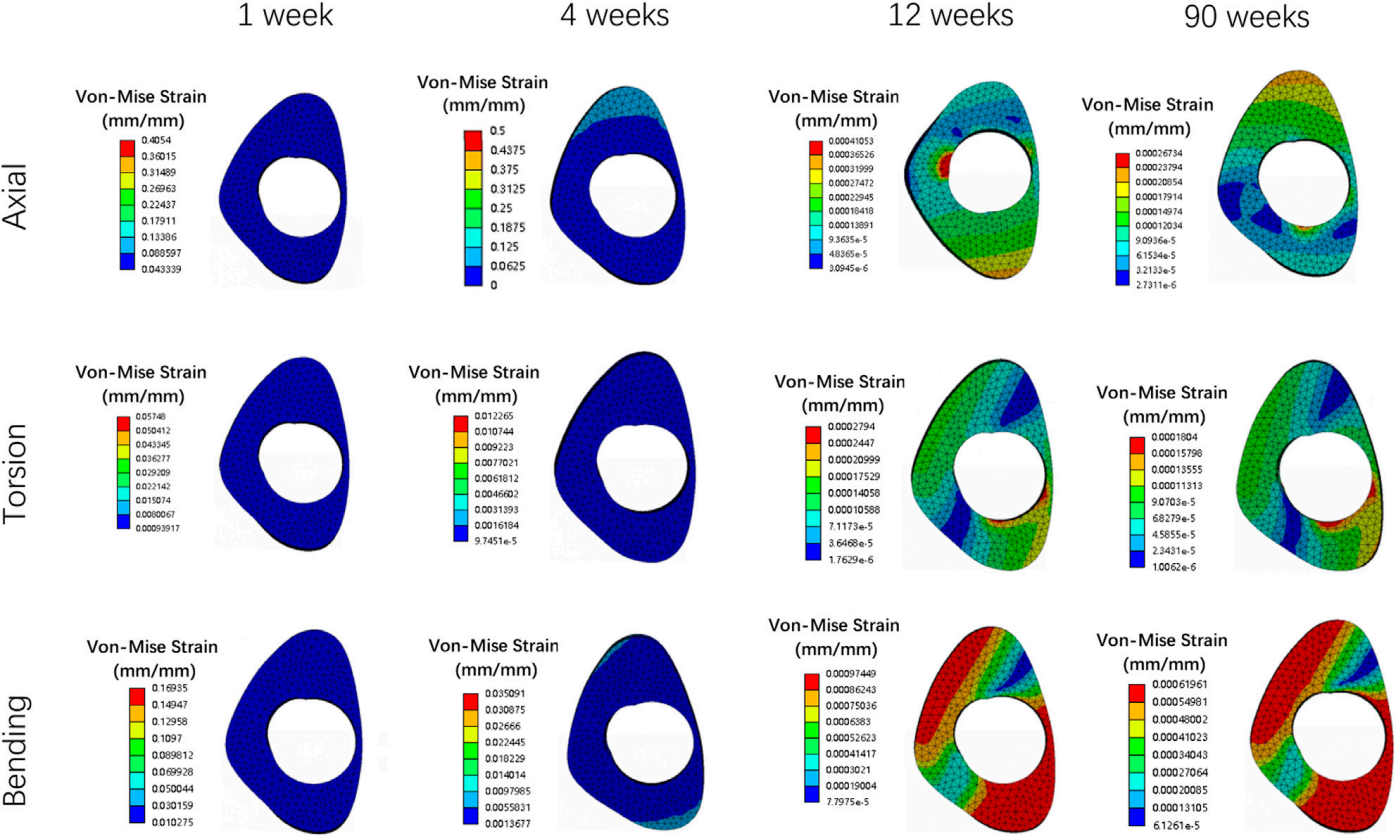
The stress cloud map of callus in all loading conditions in different stages of bone healing.

**FIGURE 6:**
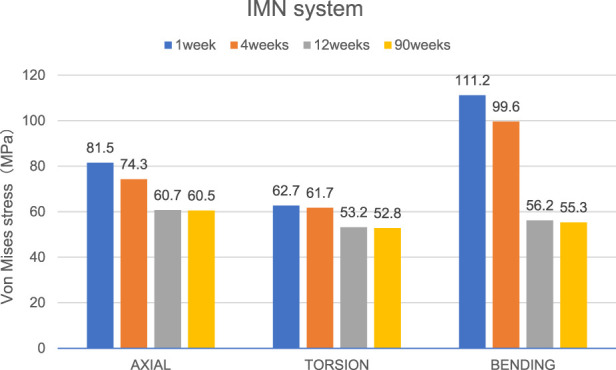
The Von Mises stress distribution on the IMN at the fracture area in all loading conditions in different stages of bone healing.

**FIGURE 7:**
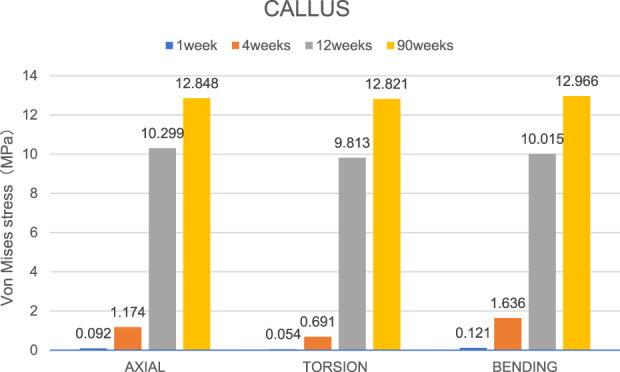
The maximal Von Mises stress distribution on the callus in all loading conditions in different stages of bone healing.

Overall stress distribution of the tibia and IMN fixation device during different stages of bone healing under varying loading conditions are shown in Figure 3. Figures 4, 5 shows the stress variation cloud map of the fracture area during bone healing. Under axial loading, in terms of the stress value of the IMN at the fracture area, at the first week after surgery, the stress value was 81.5 Mpa. At the fourth week after surgery, the stress value was 74.3 Mpa. As bone healing progresses, the stress value continuously decreases and gradually stabilizes at week 12, with a stress value of 60.7 Mpa, and 60.5 Mpa at 90th week. In terms of the stress value of the callus, at the first week after surgery, the maximum stress value of the callus is 0.092 Mpa, and 1.174 Mpa at the fourth week. The stress value of the callus gradually increases, up to 10.299 Mpa at 12 weeks after surgery. Finally, in the 90th week, the value is 12.848 Mpa. Under torsional loading, regarding the stress value of the IMN at the fracture area, the stress value was 62.7 Mpa at the first week after surgery. At the fourth week after surgery, the stress value was 61.7 Mpa. Similar to axial loading, the stress value continuously decreases and gradually stabilizes at week 12, with a stress value of 53.2 Mpa, and 52.8 Mpa at 90th week (Figure 6). About the callus, the maximum stress value of the callus is 0.054 Mpa at the first week after surgery, and 0.691 Mpa at the fourth week. As the process of the bone healing, the stress value of the callus gradually increases, up to 9.813 Mpa at 12 weeks after surgery. Finally, in the 90th week, the value is 12.821 Mpa. Under four-point bending loading, the maximum stress value of the fixation system was 111.2 Mpa at the first week after surgery, and the stress value was 99.6 Mpa at the fourth week after surgery. The maximum stress value continuously decreases and gradually stabilizes at week 12, with a stress value of 56.2 Mpa, and 55.3 Mpa at 90th week. About the callus, the maximum stress value of the callus is 0.121 Mpa at the first week, 1.636 Mpa at the fourth week, 10.015 Mpa at 12 weeks and 12.966 Mpa at the 90th week after surgery (Figure 7).

### Deformation and displacement of the callus

**FIGURE 8:**
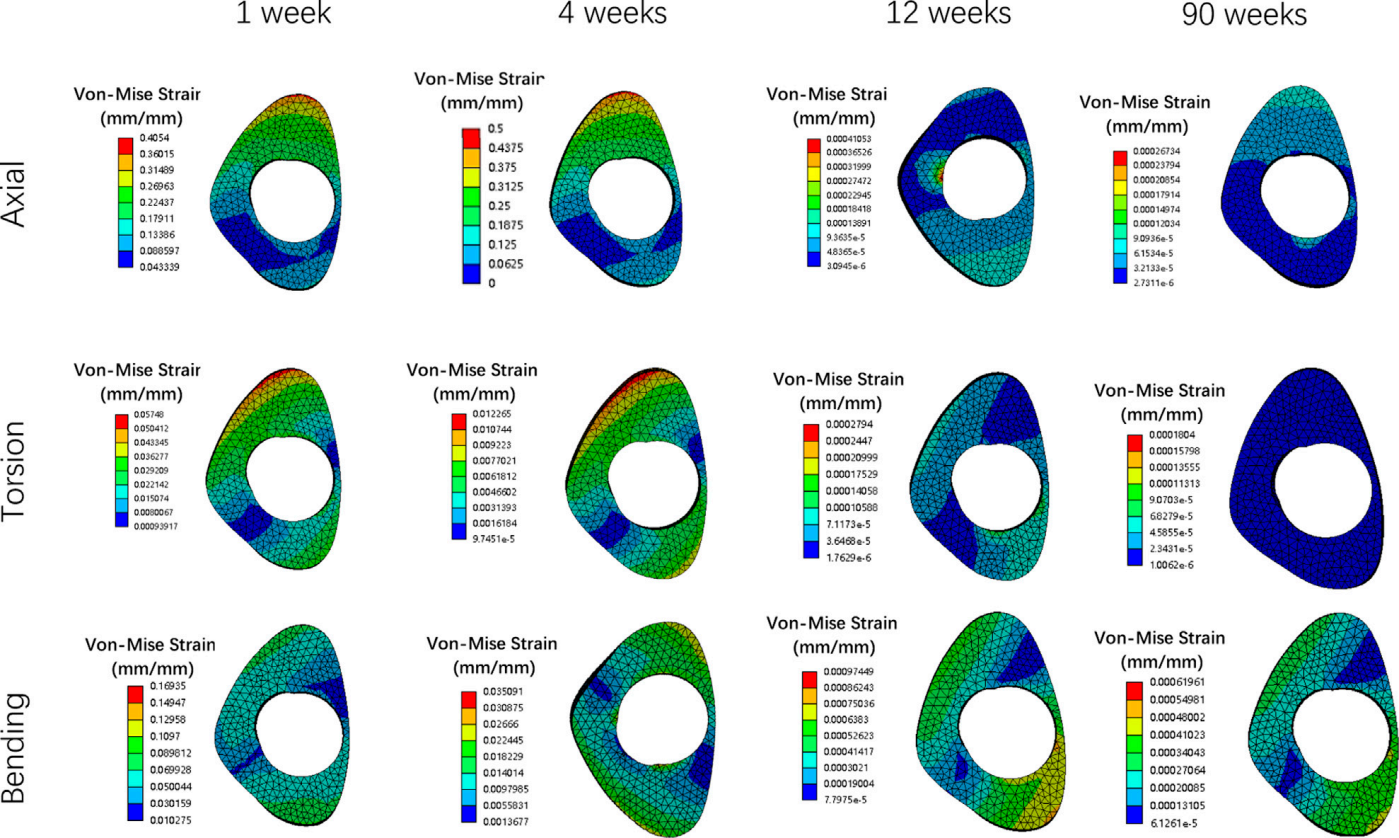
The deformation cloud map of callus in all loading conditions in different stages of bone healing.

**FIGURE 9:**
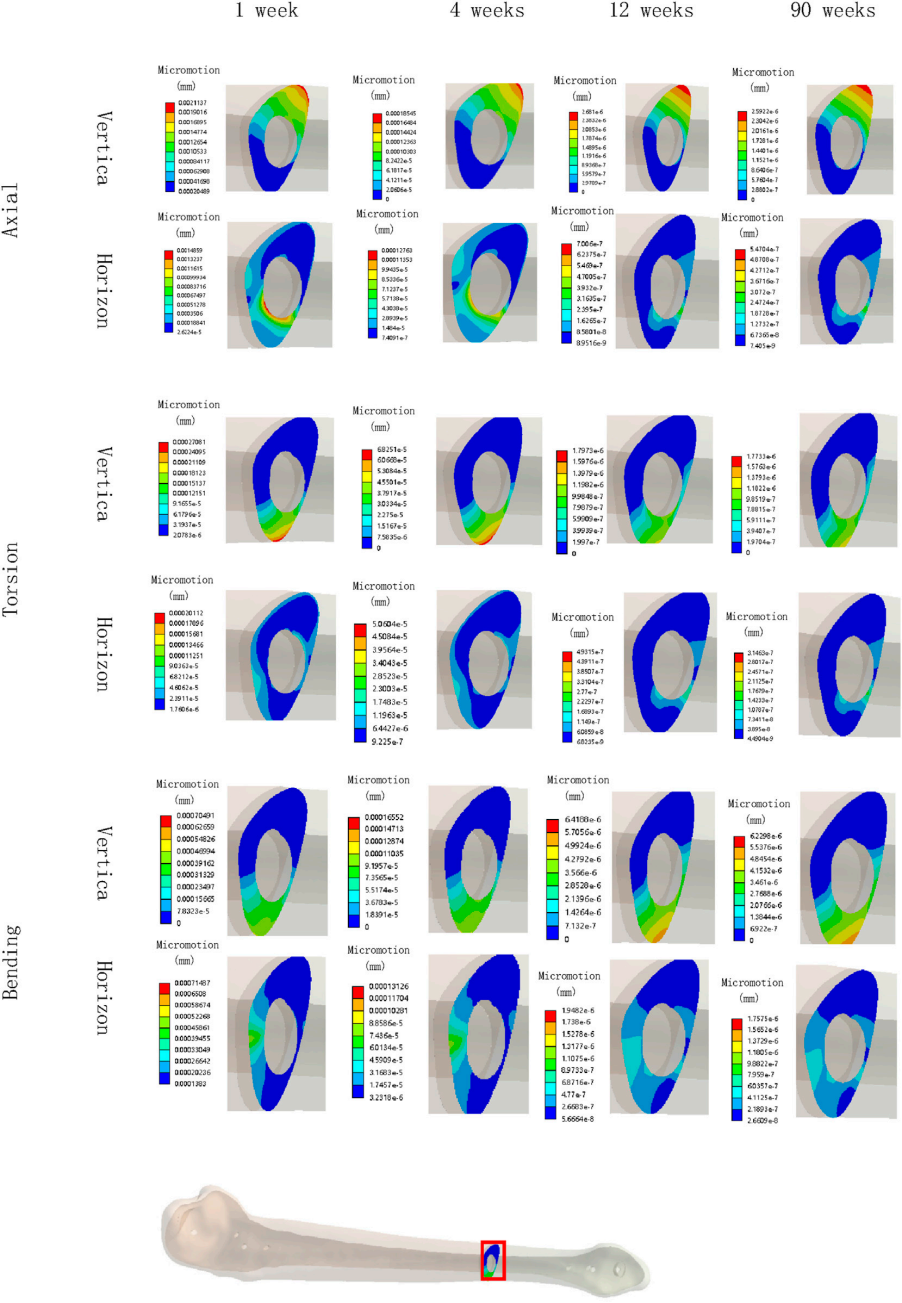
The displacement cloud map of the callus in all loading conditions in different stages of bone healing.

Figures 8, 9 illustrate the deformation and the displacement stress map of the callus. Regarding the deformation of callus, under axial loading, the deformation value of the callus is 0.013122 mm/mm at the first week, 0.0090411 mm/mm at the fourth week, 0.0011041 mm/mm at 12 weeks and 0.00078675 mm/mm at the 90th week after surgery. Under torsional loading, the deformation value of the callus is 0.0077025 mm/mm at the first week, 0.0053277 mm/mm at the fourth week, 0.0010049 mm/mm at 12 weeks and 0.0085493 mm/mm at the 90th week after surgery. Under four-point bending loading, the deformation value of the callus is 0.017163 mm/mm at the first week, 0.012606 mm/mm at the fourth week, 0.0011187 mm/mm at 12 weeks and 0.00086015 mm/mm at the 90th week after surgery. In terms of the displacement of the callus, under axial loading, the displacement value of the callus is 0.016545 mm at the first week, 0.015378 mm at the fourth week, 0.00064873 mm at 12 weeks and 0.00065839 mm at the 90th week after surgery. Under torsional loading, the displacement value of the callus is 0.0064907 mm at the first week, 0.0053567 mm at the fourth week, 0.00079406 mm at 12 weeks and 0.00077302 mm at the 90th week after surgery. Under four-point bending loading, the displacement value of the callus is 0.014586 mm at the first week, 0.012627 mm at the fourth week, 0.0015829 mm at 12 weeks and 0.0015999 mm at the 90th week after surgery. Throughout the process of bone healing, the vertical displacement distance is greater than the horizontal displacement distance for almost all loading conditions, and the horizontal displacement distance of the callus can be ignored. It is apparent that callus displacement and deformation gradually decrease. In the first week to the fourth week post-fracture, the deformation and displacement of the callus are relatively obvious. However, by the 12th week, the above indicators had significantly decreased, and there is little difference between the 12th and 90th weeks.

## Discussion

Fractures occurring in the middle and lower third of the tibia are common lower limb fractures, accounting for approximately 50% of all tibial fractures (Sobol et al., 2018). These fractures often result from high-energy injuries and typically involve complex comminution with displaced fractured fragments, necessitating surgical intervention to achieve anatomical reduction and prevent severe complications. Among the various surgical approaches, the use of IMN is a commonly employed method (Chen et al., 2018).

Currently, the common methods for surgeons to examine the degree of bone healing included X-rays and computed tomography (CT) (Burkus et al., 2017). The presence of three cortical bridging calluses among the four cortices on anteroposterior and lateral X-rays of the tibia is generally regarded as indicative of healed fractures (Salih et al., 2015). However, radiographic evidence is an indirect method for fusion assessment and may not adequately reflect the extent of bone healing, and the radiographic images are static observations of the bone healing site and cannot provide information about the fusion integrity during various motions of tibia. Besides, frequent postoperative X-rays and CT subject patients to unnecessary radiation exposure, in addition to imposing financial and time burdens. X-rays only provide morphological information, lacking the ability to directly monitor stress at the fracture area and accurately identify cartilage formation in the early stages after surgery. Finally, X-ray imaging cannot determine the trend and distribution of stress changes after surgery, limiting the potential for systematic and in-depth research.

To address these limitations, our research focuses on monitoring the stress, deformation, and displacement values of the fracture area in the fixation system and surrounding areas of the fracture to determine the bone healing process. Based on the changes in stress at the fracture area of the IMN, we can place stress sensor at fracture area to monitor the stress value to determine the stage of bone healing, and based on the displacement value of the callus, we also can place a displacement sensor at the callus area to monitor its displacement values to determine bone healing status, which replacing the need for frequent X-rays and CT in the later stages. This research aims to lay the groundwork and provide insights for the future development of IMN with stress measurement sensors, thereby offering valuable guidance for postoperative patient rehabilitation. In numerous studies both domestically and internationally, stress sensors have also been used to monitor the process of bone healing, further enhancing the position of intelligent sensing detection in bone healing and providing a solid foundation for subsequent intelligent monitoring (Jeyaraman et al., 2023; Viswanathan et al., 2023; Wolynski et al., 2019; Pelham et al., 2017; Fernández-Gorgojo et al., 2022; Najafzadeh et al., 2020; Salas-Gómez et al., 2024; Anwar et al., 2024; Wang et al., 2024).

FE analysis has been employed in this study to predict the influence of specific factors in the system and gain a better understanding of the geometrical effects (Damron et al., 2016). The FE analysis software was utilized to evaluate extra-articular distal tibial fractures based on a 3D finite element model, aiming to investigate the biomechanical distribution of the fracture area.

### The stress value changes at fracture area

Von Mises stress is widely used in finite element studies to assess the risk associated with orthopedic implant designs and failures (Shen et al., 2022). Since stress and strain are directly related, this study primarily focused on analyzing the stress changes of the IMN at the fracture area and the callus in all loading conditions in different bone healing stages.

During the process of bone healing, the stress values at fracture area constantly change under different loading conditions. From first week after surgery until the fourth week, the stress value of the IMN at the fracture area gradually decreases from its peak value. The reason is that as bone healing progresses, osteoblasts gradually appear and callus forms, and the stress borne by the lower limbs gradually shifts from the IMN at the fracture area to the callus at the fracture end. Therefore, while the stress of IMN at the fracture area decreases, the stress value at the callus gradually increases. Due to the small time span at this stage and the incomplete formation of a skeletal connection structure, the range of stress changes is not significant. From the fourth week to the 12th week, the callus at the fracture end is significantly formed, the continuity of the bone cortex is restored, the connection strength of the fracture end is enhanced, and the stress on the lower limb is quickly transferred from the IMN at the fracture area to the callus at the fracture end, resulting in a significant decrease in the stress value of the IMN, while the stress value at the callus is significantly increased. From the 12th to the 90th week, due to the basic formation of bone cortex, there is no significant change in stress values around the fracture area.

Based on the stress distribution obtained from the finite element model analysis, in order to reduce the need for frequent postoperative X-ray examinations, stress sensors can be placed at the IMN at the fracture area. By monitoring stress sensor values in real-time at different time points after surgery, the bone healing process in patients can be diagnosed, enabling a more accurate and convenient assessment of bone healing status post-surgery. When a significant decrease in stress value is observed at this monitoring point, it can indicate the initial healing of the fracture.

### The deformation value changes of the callus

As bone healing progresses, the deformation value of the callus is constantly changing. From the first to fourth week after surgery, due to the gradual osteogenesis of osteoblasts and the continuity of bone cortex, the strength of the callus at the fracture end gradually increases, and its deformation value also decreases. However, due to the weak osteogenic ability, incomplete osteogenesis, and short time period of this segment, the changes in stress values at this stage are not significant, and the degree of reduction in deformation values is also not significant. From the fourth week to the 12th week, bone healing enters a critical osteogenic period, with intact and continuous bone cortex and significantly increased callus strength, while wrapping around the fracture end. Therefore, during this process, the deformation value of the callus is significantly reduced. From the 12th week to the 90th week, the process of bone remodeling begins. As the formation of the callus has been basically completed, there will be no significant change in the stress at the callus during this process, and the deformation value of the callus will not change significantly. This means the initial completion of bone healing in 12th week.

### The displacement values change of the callus

Similar to the deformation value of callus, the displacement value of the callus also changes continuously with the process of bone healing. The displacement of the callus is divided into vertical displacement and horizontal displacement. Due to the influence of gravity, the vertical displacement changes more significantly, while the horizontal displacement of the callus remains almost unchanged. From the first week to the fourth week after surgery, due to the initial formation of the callus and the poor continuity of the bone cortex, the strength of the callus is relatively small, and the vertical displacement value is relatively large under the action of lower limb gravity. Due to the lack of obvious osteogenic effect, there is no significant change in the strength of the callus at this stage, so the displacement value of the callus is relatively large and the change value is relatively small. From the fourth week to the 12th week after surgery, the callus at the fracture end was significantly formed, and the continuity of the bone cortex gradually restored to integrity. As a result, the strength of the callus was greatly increased, and under the same gravity, the displacement value significantly decreased and the change value is relatively large. From the 12th to the 90th week after surgery, due to the basic completion of bone formation, the strength of the callus has reached its maximum value and is in a stable state. At this time, under the same gravity, the displacement value of the callus has hardly changed, and the entire tibia and fixation device have reached a balanced state. This also indicates that fracture healing has been preliminarily completed at this stage.

Based on the displacement values change of the callus obtained from the finite element model analysis, we can install displacement sensors at the callus of the fracture end to determine the state of bone healing based on the magnitude and changes of the values displayed by the displacement sensors, thus enabling a more accurate and convenient assessment of bone healing status after surgery. When the displacement value suddenly decreases to a stable value, it can indicate the initial healing of the fracture. This can also to some extent compensate for the shortcomings of radiological examinations.

## Limitations

This study possesses several limitations that should be acknowledged. Firstly, the finite element analysis model design may not completely capture the actual changes occurring in bone and the IMN after fractures, and the selected parameters may not fully simulate all daily-life activities. Additionally, the model was simplified and did not include muscles and the fibula, potentially affecting the load distribution on the implants. For future studies, it is recommended to develop more anatomically accurate finite element models that account for the presence of muscles and the fibula.

Secondly, this study solely focused on transverse fractures in the lower 1/3 of the tibia, therefore, the stress changes observed may not reflect the patterns seen in other types of tibial fractures or fractures occurring in different body areas, such as upper limb fractures, hip fractures, spinal fractures, and others. Future research should include verification models for various internal fixation devices, such as external fixation and locking plate.

Lastly, this study employed data from a single healthy adult tibia for finite element modeling and did not incorporate statistical considerations. Consequently, the findings may possess a degree of randomness. Further investigations should involve multiple participants and the development of *in vitro* biomechanical models for monitoring bone healing at different stages to validate the conclusions derived from finite element analysis. This would establish a comprehensive research system and theoretical framework encompassing finite element analysis, biomechanics, animal experiments, and *in vivo* studies.

## Conclusion

This study utilized FE analysis to evaluate stress, deformation and displacement between the IMN and bone during the healing process of tibial fractures in four stages. Based on the changes in stress at the fracture area of the IMN and the displacement value of the callus, we can place a stress sensor at the fracture area of the IMN and a displacement sensor at the callus area. By combining these aspects, the degree of bone healing can be assessed. This research enables orthopedic doctors to monitor the progression of fracture healing without relying solely on imaging examinations. Furthermore, it aids in guiding patients to undergo appropriate rehabilitation training for better recovery.

## Data Availability

The original contributions presented in the study are included in the article/supplementary material, further inquiries can be directed to the corresponding author.

## Abbreviations

IMN, Intramedullary nails; FE, Finite element; CT, computed tomography.
